# Barriers and Facilitators for Implementation of Individualized Fire Safety (IFS) in Sweden

**DOI:** 10.1007/s10694-021-01138-6

**Published:** 2021-05-26

**Authors:** Johanna Gustavsson, Gunilla Carlsson, Margaret S. McNamee

**Affiliations:** 1 Risk and Environmental Studies, Centre for Societal Risk Research, Karlstads Universitet, Universitetsgatan 2, 651 88 Karlstad, Sweden; 2 Active and Healthy Ageing Research Group, Department of Health Sciences, HSC, Lunds Universitet, Margaretavägen 1 B, 22240 Lund, Sweden; 3 Division of Fire Safety Engineering, Lund University, John Ericssons väg 1, 221 00 Lund, Sweden

**Keywords:** Personalized fire safety, Vision zero, CFIR, Consolidated Framework for Implementation Research, Implementation research

## Abstract

In 2010, the Swedish Civil Contingencies Agency (MSB) announced a “vision zero” of zero fire deaths in Sweden by 2050. Studies into fire deaths have identified that certain risk groups, including but not limited to older people, are overrepresented in fire death statistics in Sweden. The MSB has developed guidelines for how individualised fire safety (IFS) can be implemented in local communities for risk groups, in support of their vision zero for fire deaths. This paper presents the results of an interview study with a selection of Swedish municipalities to further explore how municipalities are working with IFS programs for community dwelling older people. The Consolidated Framework for Implementation Research has been used to analyse data developed through semi-structured interviews, from an analysis of the delegation of authority from MSB to local level and assessment of secondary documentation from national, regional and local organisations. The analysis has identified that IFS has, indeed, been implemented to varying degrees in Sweden, but that there are both facilitators and barriers which can be further leveraged to improve the implementation of IFS in the future.

## Introduction

Despite a significant decrease in the number of fire deaths in Sweden since the 1950’s [1], in recent years the number of fire related fatalities has been relatively constant at approximately 100 per year. Of those who die in fires each year, approximately 75% die in their homes [2]. One group that appears to be particularly at risk is older people [3, 4]. Given predictions of an aging society [5], it is reasonable to expect the number of fire fatalities to grow rather than decline in this group.

Risk factors that have been identified for older people include but are not limited to, a decreased ability to prevent a fire incident, reduced ability to respond to a fire incident and a reduced ability to remove themselves from the fire scene, circumstances which can all exacerbate the situation leading to a fatality [6]. Reduced function can be related to a variety of diseases, such as cardiovascular disease and impaired immune system [7, 8], but also functional limitations associated with aging, such as reduced physical strength and stamina, cognitive challenges, hearing difficulty and reduced sight [9]. Further, living conditions, such as living alone, increase the risk [10]. Programs for individualised fire safety (IFS) presented in the literature usually include preventative home visit, aiming to e.g. identify, assess and evaluate needs for fire safety measures [11–13]. Promising results are presented, however, the effects on injury reduction are inconclusive [14–16]. Numerous technical systems designed to reduce the risk of fires are compromised due to the natural physical and psychological challenges associate with aging, e.g. there are indications that traditional domestic smoke alarms may have reduced efficacy in this group [17, 18]. Exacerbating this situation, Sweden has an age-in-place principle, which has resulted in a large proportion of frail older people living alone with support from home care rather than in nursing facilities [19].

As early as the 1990’s, the Swedish parliament as one of the first countries in Europe, adopted a vision of zero deaths in road traffic [20–22]. Although the question of success or otherwise of such a vision is naturally subjective and complex, numerous studies have typically found the vision zero for road traffic deaths to be largely successful [23–26]. In Sweden, the “Vision Zero” terminology has consequently spread to other policy areas, ranging from suicide prevention to drug use in schools [27]. In 2010, the Swedish Civil Contingencies Agency (MSB) issued a Vision Zero concerning fire deaths for Sweden [28]. This adoption has resulted in numerous initiatives to understand the question of who dies in fires in Sweden, and how these might be prevented [1–4, 29–32]. Identification of the fact that most fire fatalities occur in homes, and that older people represent a particularly vulnerable risk group, lead MSB to issue public guidelines to the Municipal Fire and Rescue Services (FRS) concerning IFS in 2013 [33], introducing a concept for systematic fire safety targeting vulnerable groups, including criteria for identification of risk individuals and their needs to reduce fire risk. Some additional details of the methodology are provided in Sect. 2.1. There are, however, significant challenges for the FRS to implement IFS in homes. In 2017, all Swedish municipalities were contacted to survey their ongoing or planned activities in relation to IFS for risk groups. In total 70% of municipalities provided answers, of which 52% reported that they were actively implementing the concept of IFS in homes for risk groups [34]. In many cases, however, free text answers indicated that the municipalities did not have a systematic approach, possibly due to the somewhat fragmented question of which local governmental agency is responsible for implementation of preventative or mitigation measures, i.e. health care, social services or the FRS. The health care and social services personnel in the municipalities regularly visit many frail older people in their homes but are lacking knowledge of fire safety. In the case of fire safety, the FRS possess the necessary expertise concerning early warning signs and technical systems to mitigate specific fire risks but does not have regular access to risk groups in their homes. Municipalities that indicated that they were not providing IFS in the home in the 2017 survey identified a number of reasons for this lack of service, e.g. lack of personnel and technical resources, unclear division of responsibilities between service providers, potentially conflicting priorities between difference providers and the challenge of collaboration across different organisations and budgets. These results are supported by the findings of Gjøsund [35] that specifically addresses the collaboration between health and social services and the FRS.

The question of IFS and its implementation is complex [36], requiring more in-depth results to analyse facilitators and potential barriers to the implementation of IFS in homes. This paper presents the results of an interview study with a selection of Swedish municipalities to further explore how municipalities are working with individualised home fire safety programs; and, if they report on-going activities in this field, to explore the extent of these activities. Ultimately, the aim of this work has been to explore barriers and facilitators for implementation of IFS interventions in Swedish municipalities as a basis for recommendations concerning said implementation. The Consolidated Framework for Implementation Research (CFIR) presented by Damschroder et al. [37], has been applied as a tool for the analysis.

## Home Care and Safety for Older Adults in Sweden

In Sweden care of the older adults is usually provided in their home by health care and social services. The responsibilities of such care is far ranging, including aspects both of medical care (e.g. dispensing of medicines), occupational and physiotherapy, nutrition, and creating a safe environment. One aspect of safety for older people is fire safety, but other aspects include, but are not limited to, fall risks. Home care and safety of the older is governed at levels:

1. *National level* At a national level the Health and Medical Services Act (SFS2017:30) defines that all citizens are entitled to good health and care, taking into account every individuals’ equal value and dignity. Little information is given in this fundamental act as to how care shall be provided and who is responsible for this provision, although regional and local government and non-government actors are identified. Similarly, the provision of societal protection is defined in the Civil Protection Act (SFS2003:778). In the case of the Civil Protection Act, however, it is clearly stated that the municipality is responsible for the provision of a FRS. At this level, the instruments of the national government are legislation, policy declarations, various financial instruments and reporting requirements or supervision of healthcare outputs. The details of the provision of care is to be developed at the regional and/or local level. Finally, the Housing Adaptation Grant Act (SFS2018:222) governs which physical features can be adapted in homes to support people to live independently in their homes, and the Patients’ Rights Act (SFS2014:821) strengthens the individual’s personal integrity and right to decide their specific needs albeit based on expert input from care givers.
2. *Regional level* The counties (often called regions) are responsible for the provision of health care in hospitals and primary outpatient care. Decisions concerning this responsibility are made by a designated County Administrative Board. The counties are not responsible for FRS as these are the responsibility of the municipality; but, in some cases collaboration between municipalities creates federations of municipalities creating something similar to counties for the FRS.
3. *Local level* The municipalities are responsible for social services including residential care of the older adults. Social service is governed by the Social Services Act (SFS2001:453) but since the early 1992’s a reform moved most home health care for older people to the local level including both home care and nursing homes. The municipalities are responsible for the provision of a functional local FRS.

**Figure 1:**
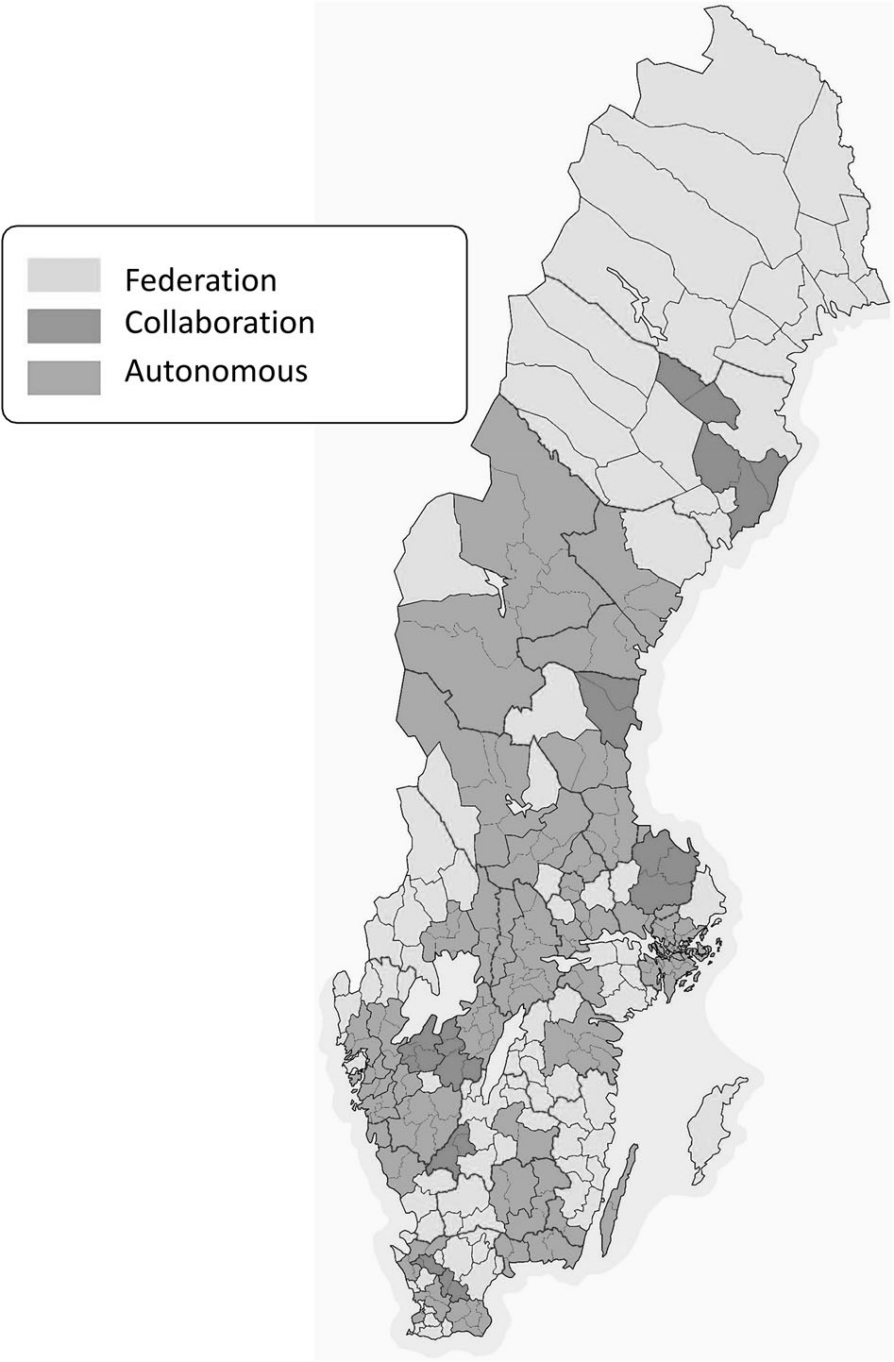
Map of Sweden showing the location of FRS federations, collaborations or autonomous municipal services. The red lines on the map show the boundaries between the various counties in Sweden and the grey lines show boundaries between the individual municipalities (color figure online)

The division of responsibilities described above is further complicated by the fact that for the sake of efficiency, the FRS has been organised into more or less strongly bound federations or collaborations, which do not necessarily match the region or county division. Figure 1 shows which municipal FRS are organised into federations, collaborations or remain entirely autonomous.

In total there are presently 21 counties in Sweden, although this number has varied over time. Each county has a board headed by a governor with the charge to supervise local government administration and to coordinate between the local and national government levels. The Government has delegated regulation of the FRS to the MSB at the national level, although the oversight role is further delegated to the County Administrative Board at the regional level. In the case of healthcare and social services the Government has delegated regulation to the National Board of Health and Welfare and the Swedish Agency for Health and Care Analysis at national level. The common interests of the municipalities and regions are in turn represented in many settings by a non-profit stakeholder organisation funded by the municipalities and regions on a proportional population basis called the Swedish Association of Local Authorities and Regions (SALAR). At the local level, the Local Government Act (SFS 2017:725) awards municipalities a high degree of autonomy expressed through their publically elected political boards. This inherent autonomy allows each municipality and region to decide on the local organisation of their services, such as fire services and healthcare. The municipalities staff the specific fire safety, healthcare and social services for the citizens. Annual planning for the services is governed by municipal business planning while in the case of the FRS there is an additional layer of planning directly to MSB at the national level which is on a triannual basis rather than an annual basis.

**Figure 2:**
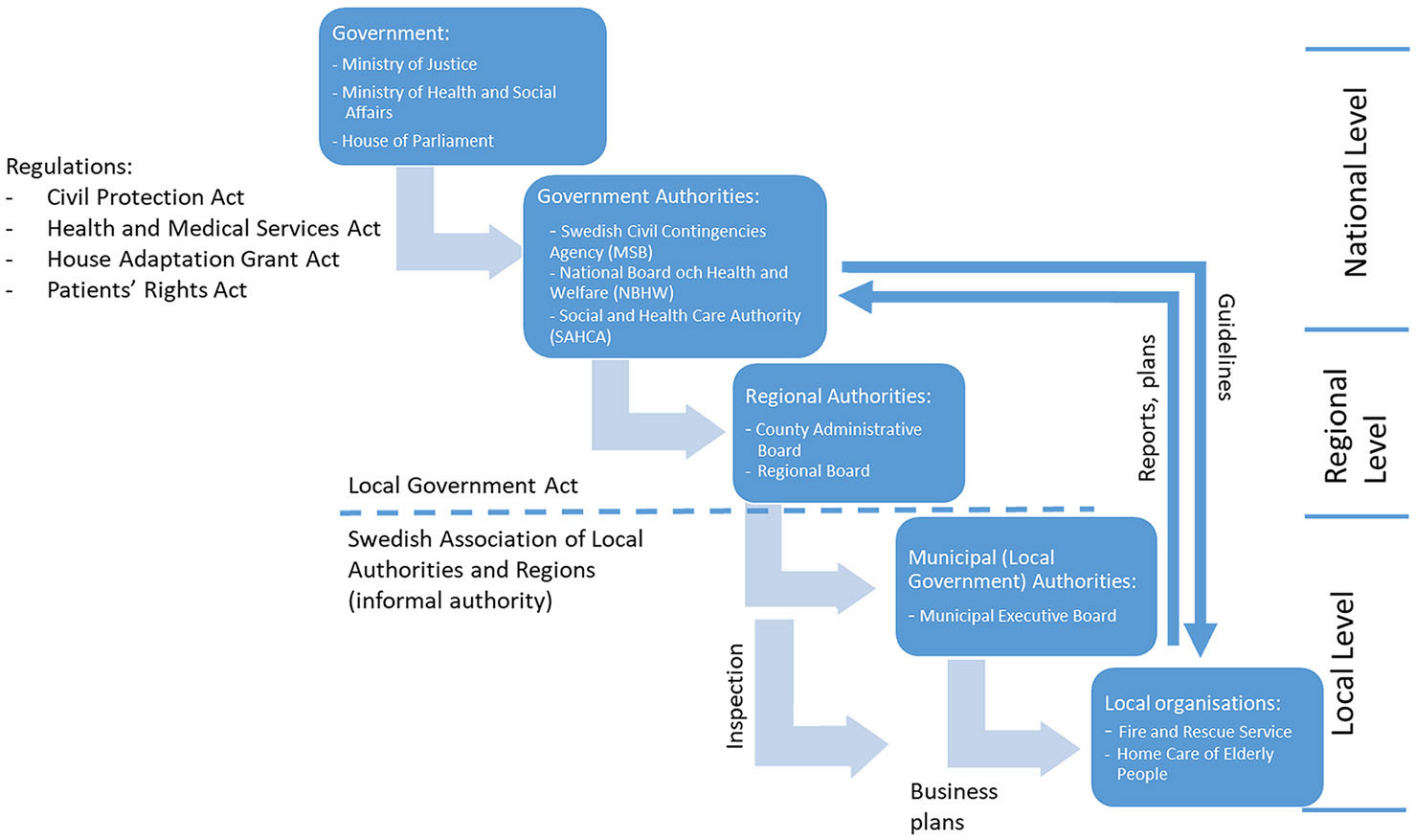
Overview of responsibilities and decision making for fire safety, healthcare and social services for Swedish older citizens

Figure 2 gives an overview of this division of responsibilities and governance of fire safety, healthcare and social services for Swedish older citizens in their homes.

### Individualised Fire Safety (IFS)

**Figure 3:**
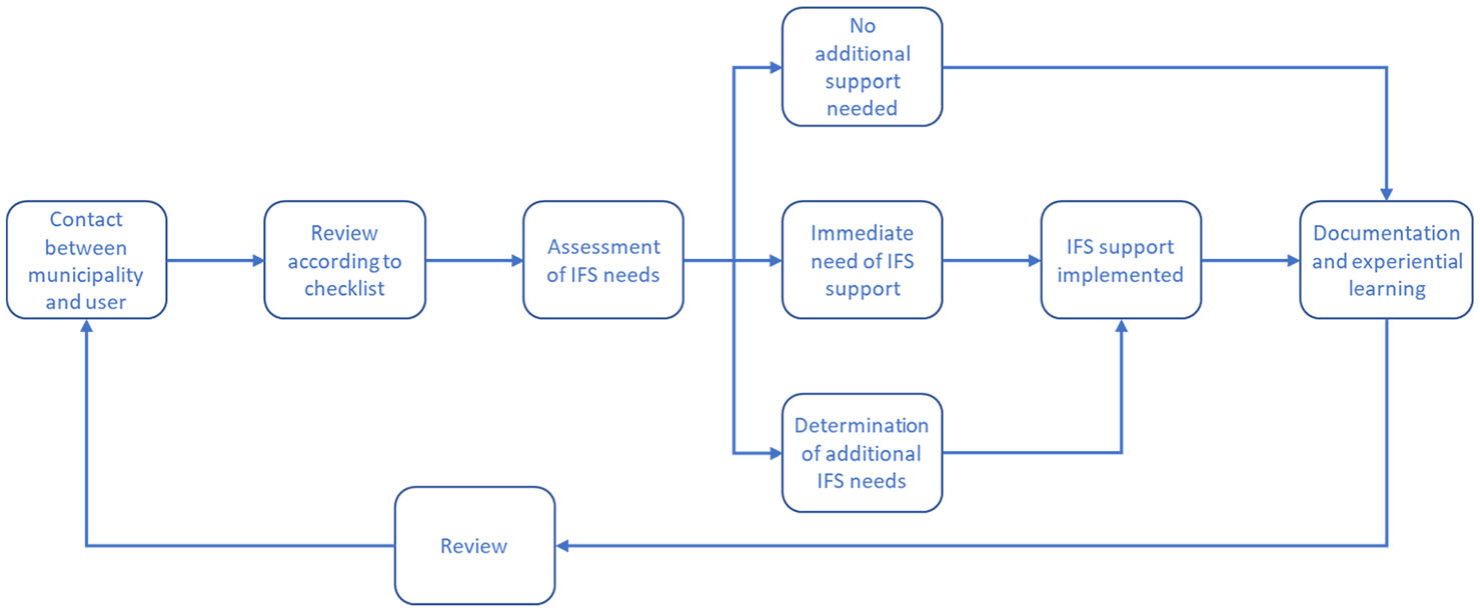
Example of process recommended by MSB. Based on Fig. 2 in reference [33]

The responsibility for IFS is placed at the municipality level. While the details of the process are left to the local municipality to determine based on their context and specific needs, examples of a basic structure for the implementation are provided based on methods which have been implemented by municipalities of varying sizes, see Fig. 1, an example implemented used by some municipalities in the south of Sweden (Fig. 3).

As mentioned in the introduction MSB issue public guidelines to the Municipal FRS concerning individualised fire safety (IFS) in 2013 [33]. IFS is not a fixed method, rather, the guideline introduces a way to systematically organize fire safety initiatives for vulnerable groups, based on the model of continuous improvement of processes, the so called PDCA model with the letters standing for Plan, Do, Check, Act [38]. It includes tools for identifying risk groups and individuals, based on whether they exhibited behaviour leading to an increased fire risk, had a limited ability to identify a fire, or had a limited ability to respond to a fire. Using these identification criteria, those in need of IFS are grouped depending on whether they are living in assisted accommodation, living in their homes (either with additional support or not) and whether they have addictions or mental health problems. Older adults, the focus of this article, are potentially represented in all four groups. The concept of IFS, is then based on an evaluation of the individual’s needs and capabilities and identification of suitable measures to reduce the fire risk, and to implement this measures. IFS promotes the FRS, in cooperation with social services, to systematically work with improving fire.

## Theoretical Framework

The foundation of the public sector, including healthcare and the FRS, is the underlying assumption that organizing welfare in a public setting is a more efficient use of collective resources that relying on the free market to produce the same services [39]. Such organisations often have a positive track record concerning the implementation of policies and services to their citizens, provided the services offered are well understood and expectations are in alignment between citizens and service providers [40]. Unfortunately, such organisations have been found to be less well equipped to respond to non-routine services, in particular to “wicked problems”, i.e. those that are complex or open-ended [40]. The question of IFS could be defined as a wicked problem due to the fact that there is no standardised solution which can be applied in all situations. Head and Alford (2015) established that while there may be no clear solution to a wicked problem, it is possible in most cases to break them down into subproblems where solutions can be sought.

Implementation of interventions and research into practical context is not an easy undertaking. Indeed, there are indications that attempts to introduce change fail in at least two-thirds of cases [41]. A wide variety of constructs have been developed to analyse the interplay between an intervention and its implementation [42]. The theoretical framework presented by Damschroder, Aron [37] has synthesised 19 underlying constructs in the Consolidated Framework for Implementation Research (CFIR) to identify key factors for implementation.

The CFIR identifies five separate dimensions, key to understanding an implementation:

1. *Intervention characteristics* This described the characteristics of the intervention itself and how these support implementation, e.g. the source of the intervention, the body of evidence to support the intervention and its quality, the advantage of the intervention relative to existing methods, the adaptability of the intervention, its scalability and complexity, the design quality and packaging and finally the cost.
2. *Outer setting* This describes the external context of the intervention, e.g. recipient needs and resources, the existence of supportive external networks, external peer pressure for change and external policies and incentives.
3. *Inner setting* This describes the internal context of the intervention, e.g. the internal organisational structure, supportive internal networks and communication, the internal culture, the implementation climate and readiness for implementation.
4. *Characteristics of individuals* This describes the ability of individual’s charged with implementing the intervention, e.g. knowledge and beliefs about the intervention, self-efficacy, the individual’s personal stage of change, loyalty of the individual towards the organisation and other personal attributes such as tolerance for change, values, capacity and competence.
5. *Process* This describes the existence of a process for the intervention, e.g. the existence of a planned process, the process for recruitment of individuals and champions, the execution of the implementation and the existence of a process for reflection and evaluation of the implementation.

In this study, the CFIR has been used as the lens for the analysis to identify what in these five dimensions and the underlying attributes offer support or barriers to broad implementation of IFS. In this way, we endeavour to break the wicked problem of IFS into its underlying parts. Since this framework was developed within the health care sector, the term patient is used in the original work by Damschroder [37]; but, for the purpose of this study, the client concept has been used instead.

## Method

For this study, a cross-sectional study-design was used and the purposeful sampling strategy was applied to obtain a maximum variation [43] in experiences of IFS. The primary and secondary data collected as part of this study was analysed using the lens of the CFIR [37]. The study was approved by the Swedish Ethical Review Authority (No. 2019-04163). This section presents the methodology in more detail as a backdrop for the later analysis of both primary and secondary data sources.

### Sample

The primary data consisted of semi-structured interviews with twelve interview participants. The majority were from the FRS. The two non-FRS were one from the municipal (pop 10 000) home services and one older person who was interviewed in conjunction with the observation of a home visit specifically after a fire in her home. See Table 1 for summary of interviews.

**Table 1:** Summary of Primary Data in Terms of Number of Interviewed Participants, County/Regional Council and Organization Where the Interview Participants was Employed and Secondary Data Included

Organisational level	Organisation	Primary data: Number of participants and type of data collection	Secondary data
National	MSB—Swedish Civil Contingencies Agency		Various guidelines Karlstads Agreement
National	Swedish Government		Laws and regulations
Municipality	Luleå autonomous FRS, Norrbotten	1 (virtual)	Checklist
County	Southern Federation (FRS), Federation Skåne North West	2* (home visit + virtual, the FRS representative in this interview was interviewed on two separate occasions) 1 (virtual)	Checklist YouTube video Project report including checklist
Municipality	Home care (social services) in Örkelljunga, Skåne	1 (virtual)	Project report including checklist
County	Federation Södertörn Fire Defence Association (FRS), Stockholm		Checklist
County	Attunda Federation, Uppland	6 (full day visit)	Guidelines
County	Federation Greater Gothenburg Area (FRS), Västra Götaland	1 (virtual)	Guidelines Checklist (Josefin data)

*One interview was conducted as part of an observation of a home visit with an older person living within the area of the Southern Federation (FRS) responsibility. The FRS representative was also interviewed virtually on a separate occasion

The sampling procedure started with identifying persons in the FRS and was based on a combination of following criteria:

1. Indication of implementation of individualised fire protection for older people from the survey performed by Jönsson and Gustavsson [34]
2. Indication of unsuccessful implementation of individualised fire protection for older people from the survey performed by Jönsson and Gustavsson [34]
3. Availability to participate in the interviews in the time frame from January to May 2020
4. Geographical spread across the country
5. Governance, representing FRS federations and autonomous municipalities

During the sampling procedure, it became clear that home care in one of the municipalities identified was heavily involved in the development and implementation of IFS, and a representative for the social service in that municipality was interviewed. All of the identified persons were contacted, received information about the study and after informed consent, chose to participate in an interview. Some aspects of the study initially planned, e.g. multiple home visits, were interrupted due to the outbreak of the covid-19 pandemic, which is why only one such visit was conducted.

The secondary data used in this study included national laws, regulations and guidelines within the field, but also data that the participants provided in conjunction with their interviews such as local checklists, an information film and project reports. Table 1 includes a summary of the municipalities included in the study, the participant distribution (relating to the primary data associated with interviews) and secondary data.

### Data Collection

The starting point for the interview guide developed was the national guidelines about IFS [33]. Themes that were addressed during the interviews were: In what way does your organization specifically work on IFS? If your organisation does not work on IFS—why not and have you previously done so? Is your organisation planning to work on IFS—what are your plans and how long have you been planning for this? Which facilitators and barriers do you and your organisation experience in working with IFS?

The interviews were, due to practical reasons, done on site as well as via link or telephone. In one organisation, six participants were interviewed across a working day on site at the FRS with one author (JG). These interviews were divided into three sessions, one was conducted with three managers together, one with two operative personnel and one with a coordinator. The final interview focused specifically on guidelines for home visits. Five of the interviews were digital, conducted via zoom by two of the authors together (GC and MM), with one participant at a time. These interviews lasted for approximately 45 min each. One researcher led the interview and the other one took detailed notes and asked supplementary questions during the interviews. The final interview was conducted in conjunction with a home visit with an older person.

### Data Analysis

For the analysis, a deductive approach was applied [44] to describe the interventions and identify barriers and facilitators to successful intervention against the backdrop of CFIR [37]. The five dimensions of CFIR and the underlying attributes of this theoretical framework guided the analysis. The reliability of the written notes were checked against the audio recorded interviews, in order to ensure that important data was not overlooked. The summaries of the interviews were entered into NVivo 12 and text was coded according to the dimensions and the underlying attributes in the CFIR [37]. During this coding process, the authors were open to introduce additional dimensions or attributes, but this was not necessary given that the framework is very accommodating.

To gain a deeper understanding of how the interventions were interpreted and implemented in the organisations, secondary data, e.g. the legal framework for the intervention, guidelines and checklists from MSB and municipalities were included (see \* MERGEFORMAT Table 1). The material provided by the interviewed organisations was integrated into the results where relevant.

Both primary and secondary data were available to all authors and the analyses was made jointly with regular data analysis meetings. The interdisciplinary research team, consisting of a nurse, a fire safety professionals and an occupational therapist, facilitated the development of different perspectives during the analysis. The results were continually discussed between the authors until agreement was reached. Illuminating quotes are presented to clarify how the empirical data supports the results. Note that all quotes have been translated from Swedish by the authors.

## Results

The results of the analysis of the primary and secondary data is presented below, using the five dimensions of the theoretical framework CFIR and their underlying attributes. Table 2 provides an overview of identified facilitators and barriers for implementation, followed by an in-depth description of them.

**Table 2:** Identified Barriers and Facilitators Presented by Dimensions and Attributes According the CFIR Framework

Dimensions	Attributes	Facilitators and barriers
Intervention characteristics	Intervention source	Need for clear guidelines locally, regionally and nationally
	Evidence strength and quality	FRS acknowledge the problem but perception of evidence strength and quality sometimes weak
	Relative advantage	Lacking due to scarce feedback on effects. IFS is an apart task for FRS
	Adaptability	High, facilitates implementation
	Trialability	High, pilots and incremental implementation is positive
	Complexity	Responsibility unclear. IFS is competing with other pressing needs. Dependent on local networks
	Design quality and packaging	Improving gradually. Success stories important
	Cost	Costs vary, depending on type. Lack of funding a significant barrier. Unclear responsibility regarding additional costs
Outer setting	Client needs and resources	Fragmented understanding and lacking resources. External networks crucial. Risk behaviour in target groups
	Cosmopolitanism	Networks difficult to withhold
	Peer pressure	Occurs, successful examples inspires
	External Policy and incentives	Legal support and policies largely in place. Somewhat contradicting policies
Inner setting	Structural characteristic	Deep-rooted organisation with set norms and values
	Networks and communications	Lack of communication and formal channels for cooperation
	Culture	Need for new tools. Lacking time. FRS not seen as a resource
	Implementation climate	Experience from social services in FRS, is helpful
	Readiness for implementation	Vary. Fires are rare events, provides a barrier. Stakeholders have different agendas
Characteristics of individuals	Knowledge and beliefs…	Perceived lack of knowledge. Experience is limited
	Self-efficacy	Prevention not internalised in the FRS role. Lack of self-efficacy. Prevention has low status
	Individual stage of change	Might not see the need. Hesitant to home visits
	Individual identification with organisation	Weak identification with prevention as a significant duty
	Other personal attributes	Strong culture norms and values provides barriers. Clear methods facilitate through increased confidence
Process	Planning	Inspiration from others. Stepwise implementation. Start small. Incorporate IFS into existing processes
	Engaging	Political and organisational support vital. Bespoke positions important. Pilot project drive implementation. Difficulty to identify key individuals in social services
	Executing	Challenging to identify, reach and motivate risk-individuals. Responsibility needed, responsibility unclear. Lack of resources. Other needs more urgent
	Reflecting and evaluating	Lack of follow up perceived as a key barrier. Prevention effect abstract

### Intervention Characteristics

#### Intervention Source

To varying degrees, all participants referred to the MSB guidelines as the source of the initiative to develop IFS. The rationale for IFS was recognised in many cases as stemming from the concept of a vision zero for fire deaths. In some cases, the municipality had developed and applied IFS interventions for older people based on the intention described in the MSB guidelines, in others there had been efforts to implement the recommendations without a specific focus on IFS but rather on mitigation of fire risks for risk groups in general. There was clear respect for the source of the intervention (i.e. MSB) although the participants reported some frustration concerning the vagueness of the central guidance documents.

#### Evidence Strength and Quality

The participants expressed an understanding that the majority of fire deaths occur in homes and that certain risk groups are over-represented in the fire death statistics, e.g. older persons. The concept of vision zero was seen to be laudable but the connection between the vision and IFS interventions were seen to be tenuous, requiring local efforts for its implementation, i.e. an understanding of local conditions and networks guided the development of the intervention. There was local resistance to the implementation of IFS in certain communities, which might indicate that evidence strength and quality is perceived as lacking. In one interview, it was acknowledged that successful implementation in one community helped to strengthen the willingness to implement in new communities. Thus, evidence could be developed through practical experience in pilot communities.

#### Relative Advantage

The participants expressed a need for results or feedback on their efforts with IFS. Since this is lacking, it can be difficult to see the relative advantage with this type of intervention compared to allocating resources to other preventative activities. The development of IFS is also a task that differs from that of traditional FRS activities, and might not align with what these organisations consider to be their role in society, and therefore not align with available resources. The fact that the FRS lack a direct line of entry to home settings reinforces the difficulty in seeing the relative advantage of working with IFS for the older persons in their homes, relative to allocating resources to, e.g. generic dialogue with pensioner organizations.

#### Adaptability

The developed methods for IFS varied from information to older people in general, to specific home visits among single individuals in risk groups, indicating a high level of adaptability. Several ways of identifying risk individuals were also described. Firstly, emergency call-outs can present encounters with risk individuals that can be offered relevant information concerning IFS. Secondly, knocking on doors in identified geographical risk areas is another option, and thirdly, cooperation with social and health services can be used to identify risk behaviour among individuals already using municipal home care.

#### Trialability

All of the participants emphasised the importance of incremental implementation and a need to be able to try different modes of intervention in order to be successful. In one of the municipalities, the FRS and the social services collaborated to develop the IFS intervention in a pilot-project, which resulted in a successful trial and political approval to continue the implementation of IFS. In another municipality within the same fire service federation, resistance had been met to the implementation of IFS historically; but the participant thought that there would be a more open attitude to future efforts given the successful implementation in a neighbouring municipality in the same fire service federation.

#### Complexity

The decision making structures described in Fig. 2 give an indication of the complexity inherent in designing and implementing IFS in any given community in Sweden. Identification of who is responsible for the implementation is at times difficult. As one participant stated, the FRS understands the technical issues, knows the fire safety problem and sees the benefit of IFS. At the same time the FRS is the smallest administrative unit in the municipality and needs to convince social services, the largest administrative unit in the municipality, of the benefit and need for IFS in competition with other pressing needs for these risk groups, e.g. basic hygiene and nutrition. Apart from providing basic care and medical assistance, there are also demands on social services for regular quality development, such as the need to alleviate fall risks. In some municipalities there was a strong working relationship between the FRS and the social services, in others this relationship was weak. The difficulty in implementation depended highly on the specific conditions and networks in the areas.

#### Design Quality and Packaging

The success of an intervention appears to be dependent on how the information is delivered, although there is no systematic information available concerning which specific types of contact work best. This translates to a lack of experiential information concerning best practices, e.g. content in written information or personal contact to at risk individuals. In one municipality, the lack of staffing resulted in identified risk individuals being provided with written information and recommendations only, while in another municipality there was an effort to book follow-up visits after a fire incident with both the social services and FRS present to provide weight to the recommendations offered.

Design quality and packaging seems to improve as IFS is implemented in one community and then moved to another for further implementation. Lessons learned and success stories from a nearby municipality appear to act as a role model.

#### Cost

Local government finances are typically strained. Several interviews revealed that there is often broad acceptance of the value of IFS, provided its implementation does not entail any additional cost. The social service sector is interested in IFS and does see the need for it; but interviewed participants from the FRS reported that lack of implementation is typically blamed on lack of resources. In one municipality the participant stated that “The common theme behind lack of implementation is that this costs work hours, and most importantly that technical solutions cost money.” Another recognised the question of which part of the municipality is responsible for the implementation of IFS is also driven by costs and budgets, “If the FRS is not able to implement IFS then there is no funding in social services for this*.”*

If funding is needed, e.g. for additional time for care professionals to deploy additional checklists, or for the installation of technical systems, this can be a significant barrier to implementation. Low cost technical systems, e.g. fire blankets, fire resistant sheets and bedding, fire resistant aprons for smokers, fire detectors and fire extinguishers must typically be paid for by the residents themselves. More expensive technical systems, e.g. stove guards can be funded via the Housing Adaptation Grant Act (SFS2018:222) or mobile water mist systems can be covered by local government funding; but this requires the client to go through an application process which may or may not be facilitated by the municipality itself. As a possible solution, one participant suggested the construction of a special fund with allocated funding from both the FRS and social services, which could cover the costs.

### Outer Setting

#### Client Needs and Resources

The division of responsibilities leads to a fragmented understanding of client needs and fragmented control of available resources. The FRS has responsibility for fire safety of citizens while social service is responsible for older people in need of social service. The introduction of new routines by the FRS for home care personnel concerning IFS, can only be implemented after agreement with the social services, it cannot be mandated by the FRS alone. The use of resources is therefore highly related to the topic of *Cosmopolitanism* according to CFIR, or established external networks and collaboration between different stakeholders providing support for risk groups.

Full understanding of the needs of individuals within identified risk groups requires a multifaceted dialogue between various stakeholders, which also involves the clients. Most older people are happy to receive help; but some, quite often those exhibiting significant risk behaviour such as heavy smoking or people with high alcohol consumption, refuse to modify their behaviour to alleviate identified risks. The Patient’s Rights Act (SFS2014:821), which supports the personal integrity and self-determination of the patient, was acknowledged by participants as something positive; but, they also expressed some frustration that it could provide a barrier to reducing risk if the person did not want any help. Indeed, participants acknowledged that the person who opens the door when they knock on doors in risk neighbourhoods are often not those most in need of help. In many cases the home care providers identify risks in patients homes, but they are unable to alleviate these risks as risk behaviour is highly entrenched, e.g. storing food in the oven, having flammable material close to source of ignition, smoking in bed and alcohol consumption.

One poignant example concerns the older person interviewed in their home who had experienced a fire. The older person explained that she had started to smoke when her husband died. She always smoke after eating and she uses smoking as a reward when she has done something positive. She was not motivated to quit and was in the habit of smoking in the kitchen sitting on a chair with a pad in front of the stove, although sometimes she smokes outdoors. One evening she fell asleep while smoking, after taking a sleeping pill and woke up from the fire alarm and pressed her service alarm. The social service could call the FRS and the social service arrived quickly and helped her out. The service personnel informed the older resident and suggested changes, but the resident must decide themselves, which changes to make to improve their personal safety.

#### Cosmopolitanism

In order to succeed with the interventions, the participants described how important it was to have well established networks and to identify the right persons in the municipalities’ home care, associations for senior citizens, or other organisations with large participation of older people. In one municipality, the FRS previously received information from social service managers about different types of fire safety measures. However, this is presently not prioritized in the political organization, and therefore no regular dialogue exists to support of the multi-facetted needs of home care patients.

In one municipality where the FRS had a dedicated senior consultant employed, collaboration with the social service managers was ongoing to ensure broad support of older people in the community. The Senior Consultant also tried to approach organisations providing accommodation aimed for people 55 years or older, and to develop a dialogue with the municipal Guardian Committee which supervise legal guardians who support citizens who for some reason are deemed unfit to make executive decisions for themselves. The Guardian Committee provides a direct point of contact with legal guardians who have regular contact with and make financial decisions for some particularly frail older persons. The existence and development of these networks and others in the community are key to the successful implementation of IFS.

#### Peer Pressure

The participants indicated an active interest in interventions taking place in neighbouring communities. Some referred to the fact that successful interventions in one municipality provided inspiration for initiating implementation of a similar intervention in another municipality. On a broader scale, the coalition of fire chiefs signing the so called Karlstad Agreement in 2016 shows a commitment to develop IFS in their communities, bolstered by peer pressure [45]. This peer pressure within the FRS community can also be used to leverage political peer pressure.

Some of the participants described that the process to implement IFS had started several years before, but that they had faced challenges to implement planned interventions, leading to a feeling of failure to support risk groups in their community. Some representatives of the FRS reflected that it is easier to implement an intervention when somebody asks for it. For example, when the home care professionals ask for support to implement IFS after they had heard about it from other municipalities they are much more receptive to input from the FRS than when the impetus for implementation comes from the FRS directly.

#### External Policy and Incentives

This attribute is a broad collection of external strategies (governmental or other) in support of the development of the intervention. In this sense, both the Karlstad Agreement [45] and local policies or guidelines in support of IFS provide a necessary backdrop to successful implementation. The vision zero policy for fire deaths as defined by MSB [27] and associated guidelines specifically for home settings [33] provide strong policy support for IFS. Legal support can also be gleaned from the Civil Protection Action (SFS2003:778), the Social Services Act (SFS2001:453), the Health and Medical Services Act (SFS2017:30), the House Adaptation Grant Act (SFS2018:222) and the Patients’ Rights Act (SFS2014:821), which all provide necessary input into the development of IFS in a community. There is significant support for implementation through this attribute.

In contrast to other policy support, client confidentiality can provide some barriers to implementation of IFS due to difficulties in identifying risk individuals. This can be circumvented by identifying risk areas in towns or cities as opposed to risk individuals. The strength of the Civil Protection Act relative to other legal protection of patient’s rights is unclear leading to additional uncertainty concerning the mandate of the FRS to act.

### Inner Setting

#### Structural Characteristics

The need for structural change in support of new practices with in the FRS, e.g. the implementation of IFS, is reinforced by identification of the need for a change agent, someone that leads the way, in certain interviews. The presence of dedicated positions helps to elevate the standing of the intervention by building organizational memory of IFS interventions. In contrast, in the absence of dedicated positions, movement of personnel from one project or position in an organization to another means a loss of organizational memory and undermines the understanding of IFS.

#### Networks and Communications

An obstacle to implementing and maintaining IFS, raised by the participants, is the lack of formal channels of communication between actors within the municipality. To successfully implement IFS, there is a need, e.g. for improved communications between FRS and the social services.

Cooperation depends on individual engagement and the participants perceive that it is up to them to create networks, both inside and outside the municipality. One example of successful networking and communication is the organisation of the much appreciated “safety days” together with pensioner organizations, where fire protection is discussed, together with other safety and health matters. The lack of established networks and lack of clear communication guidelines can be a hinder to successful implementation of IFS.

#### Culture

The FRS have a long and proud history, and the norms and values that have developed over time can be difficult to change, which means that there can be some internal resistance to preventative safety activities in general and IFS specifically. As one participant from the FRS noted “[The FRS] is a corp that has not been reformed. There is a strong professional trade union. The firefighters typically unite against change*.”* Staff turnover is low and personnel are quickly socialised according to existing norms and values. Another participant from the FRS noted that *“*those who are new to the profession are rapidly indoctrinated […] and it is a major challenge to break established patterns of behaviour.” A cultural development to expand the traditional role of the FRS to include preventative activities such as IFS has been occurring over the past decade or more, but cultural barriers to preventative activities may still exist and potentially impact on the implementation of IFS.

#### Implementation Climate

The participants expressed a view that IFS is not implemented as it ought to be and acknowledged the need for tools to increase fire prevention for risk groups. However, it might not be possible for all stakeholders to prioritise fire prevention. The social services often lack the time to e.g. identify risk individuals; and there is no clear culture of dialogue with the FRS, so they are not considered a resource for the social services to draw on. One way to bridge the gap between FRS and social services can be by having people within the FRS with educational background or experience from social services or health care. At the same time, if cooperation is dependent on a specific individual this can be a weak link in implementation.

#### Readiness for Implementation

Even though there is an awareness of the problem within the FRS, prevalence of fatal fires are low and the stakeholders might not perceive the situation as intolerable. A rare firsthand experience contributes to not perceiving the problem as urgent, which can hinder implementation.

Further, there is a perception that the agenda of key stakeholders do not align, and that it is difficult to include all perspectives and needs in home care. From the perspective of the FRS it is reasonable to expect the social services to have a checklist for fire prevention, but from the perspective of the social services this is not necessarily a prioritised task. As one participant stated “It is difficult to see all perspectives, my priorities are closest to heart.”

### Characteristics of Individuals

#### Knowledge and Beliefs About the Intervention

IFS has been a topic of interest for approximately seven years in Sweden. The Local Government Act (SFS 2017:725) means that each municipality has a mandate to determine and prioritize many activities at a local level. In one interview it was suggested that the FRS needs to improve their knowledge of home care in order to be able to tailor IFS to the actual situation in the homes of risk individuals. Many FRS have limited experience of preventative fire safety or home visits. The development of guidelines in some municipalities is helping to alleviate some of the problems of knowledge and understanding of the intervention, which can also have a positive impact on beliefs concerning the intervention. As one participant stated, “It is important to scale down ambitions to a relatively low level so that they will be doable and realistic.” Central coordination is necessary, as are local champions to ensure implementation.

#### Self-efficacy

Putting out fires and assisting at traffic accident sites are looked upon as the primary process for the FRS, and the interviews show that preventative work is considered more of a complementary process. The operative rescue service personnel might not see preventative work as a part of their role, and it can be a task that they feel less equipped to handle. This lack of self-efficacy regarding the ability to take an active role in prevention is highlighted as an obstacle, and an area where confidence and knowledge need to improve. Participants explained that firefighters are also indoctrinated in the need to work in groups or “squads”. Preventative work, such as the implementation of IFS, is typically done by single change agents, which can be seen to be in conflict with the group imperatives. Preventative positions have low status, leading lone firefighters in this field to be potentially uncertain of their role both in society and in their organisations. As one participant stated: *“*Personnel de-value themselves. Alone is uncertain. They do not understand what they symbolize and what they can achieve. They do not understand their own power, what they represent and what they can influence. In contrast to the police, where police just go in, throw themselves into situations, firefighters hesitate. Firefighters are uncertain of their role if there is no fire.”

#### Individual Stage of Change

Firefighters are sometimes hesitant about conducting home visits. They do not see the need in their context. Coupled to the fact that there is no immediate feedback concerning the success of the action, i.e. no fire is extinguished, no-one is obviously saved. There is a need to reinforce the value of preventative activities. Individuals who see the value of preventing a fire as opposed to extinguishing the fire are typically further along on the path to understanding the need for changes in the way we create fire safety for risk groups. In cases where there is a dedicated position responsible for the implementation of IFS or working with risk groups it is more common that the individual responsible for the implementation of IFS has a well-developed stage of change.

#### Individual Identification with Organization

As stated previously, the firefighter union is typically strong and participants reported that firefighters are rapidly indoctrinated into existing culture. The individuals typically identify strongly with their colleagues and their team or squad members rather than necessarily with their organization. In this case, backing from senior management is important to break potential informal structures in the organisation and strengthen the organisation itself. Individuals that are a part of the core management are more likely to identify with the organisation than operative personnel. Therefore, the creation of bespoke positions within the organisation with a clear mandate to work on IFS increases the likelihood that individuals will identify with the priorities of the organisation rather than that of individual teams or informal groups within the organisation.

#### Other Personal Attributes

Several participants pointed out that an inherent difficulty with IFS is identifying and reaching risk-individuals. When identified, the challenge is reaching risk individuals and motivating them to receive advice and make changes to improve fire safety. When knocking on doors, there is a perception that risk individuals are not the ones that are willing to open and receive advice. There is often a certain amount of resistance to approaching the public about risk behaviour. In one interview it was stated that “In the beginning I found it difficult to know what to say so that people we approached would not react in a defensive manner, that they don’t appreciate someone ringing on their door and complaining about their behaviour.” Once the participants had developed a methodology, they felt that the visits were typically seen as something positive by the residents. They emphasised the importance of, e.g. having staff that could communicate in foreign languages when approaching immigrants.

### Process

#### Planning

The IFS intervention is more of a concept than a fixed method. This opens up for innovation within the concept, and the organizations need to develop the practical procedures. As part of this process, the FRS often look at how others have made similar interventions and draw inspiration for previous experiences. The planning process is supported by peer recommendations, referring to the success of others promotes implementation which is an advantage in seeking acceptance within the organization. Through stepwise implementation, and at the same time pointing to the success of others, implementation is facilitated.

All of the participants emphasised how the heavy workload within the health care organization affects implementation. Despite being interested, working with IFS tends to drown in day-to-day tasks. In one case it was stated that “The home care staff think that it has been interesting to work with the question of IFS but the organization needs to include this work as part of the planned activities, otherwise it tends not to happen.”

It is important to recognise this need and incorporate IFS into other existing processes. In one case the implementation into existing routines had been recognized: “We have recently finished a pilot project and will begin working with IFS outside of the project format. We have developed a strategy, the home care-givers have a checklist for biennial risk rounds (scheduled in April and October each year). This timing has been chosen to suit when two other checklists are followed (one for occupational risks and one for cognitive ability).”

When combining IFS with other quality and prevention work, it becomes time effective and easily remembered. Additionally, the addressed matters can be linked to the same problems, e.g. identifying and mitigating initial memory loss problems, decreases the risk of fire as well as malnutrition and fall risk.

#### Engaging

The interviews often highlighted the importance of attracting and involving appropriate individuals in the implementation. Without sufficient political and organisational buy-in for the idea of IFS there would be no real momentum behind its implementation. Some participants talked about the need for champions and/or bespoke positions within the organisation rather than projects that come and go depending on short term priorities.

In one municipality, a pilot project concerning IFS had been tried previously but was found to succeed only when the right individuals from both the FRS and social services connected and could drive the implementation from various parts within the municipality. Once a successful outcome had been achieved, these committed individuals could provide a basis for engaging further decision makers to ensure that the project became institutionalised by creating routines.

Similarly, in those cases where municipalities described challenges to the implementation of IFS this was typically due to the difficulty of identifying key individuals and convincing them of the need for its implementation. Results show that there is often an officially appointed person leading the implementation of IFS, even though this person might also have other responsibilities, and divide their time between various tasks.

#### Executing

Execution of IFS is often a question of responsibility. Who is responsible, the FRS, social services or the individual? As one participant stated “When we consider individual houses, the regulations say that fire safety is the responsibility of the home owner. Should we charge a person with dementia because they do not have sufficient fire safety? We have considered it but it does seem inhuman given the situation.”

Some of the FRS interpreted it as their responsibility to organize the IFS within their own organization, whereas in one organization it was decided that the home care would carry out the IFS when the intervention was fully developed but that it would be developed jointly by the FRS and social services together.

If the FRS or social services take responsibility there is a risk that cases remain unresolved due to lack of time. Prioritisation is difficult when basic health and safety needs must be prioritized over the risk of relatively rare fire events. Prioritisation is also continually changing. As one participant noted, they were in the starting blocks for implementing IFS when COVID-19 arrived and used up all available resources.

In the researched organisations, the length of the decision process needed for execution varied. Independent of whether the organisation was small or consisted of several municipalities, execution of IFS typically started small, e.g. in one geographical area. Implementation in small scale made it easier to make the decisions and involve stakeholders, and lessons learnt in a limited setting could then be translated into a larger scale implementation.

#### Reflecting and Evaluating

The results clearly show that when it comes to IFS, a full feedback loop is seldom in place. This is true both at the organisational and case specific level. One reason is lack of time, but there is also a lack of recognition of the importance of reflection and evaluation. When personnel are few and tasks are many, there is seldom room to take the time to reflect on or evaluate various interventions. In particular, very little time appears to be allocated to return visits to investigate whether recommended IFS has actually been put into practice.

The lack of follow up on preventative measures is mentioned as a key obstacle for the implementation. Preventative work is perceived as abstract, as it is hard to see an immediate effect, and compared to the core task of the FRS, like putting out fires, the effects are vague. The need for follow ups as a way to increase motivation for prevention is highlighted. Further, it is difficult to measure the effect of implementation which discourages introspection and evaluation.

## Discussion

In this study we have explored the barriers and facilitators for implementation of IFS interventions in Swedish municipalities, using the CFIR as a tool for analysis. Individualised fire safety has been a topic of interest from a national level for a relatively short time period, and as each municipality has the mandate to determine and prioritize activities at a local level, it has far from reached full implementation as yet. The fact that IFS is not a fixed intervention, leaves room for a high degree of innovation, but also some room for frustration due to uncertainty concerning best practices. In this study, the results show that the general features of the interventions can be grouped into three main types, indicating a high level of adaptability. The three types of interventions focus on (1) the single individuals at risk, (2) identified groups at risk or (3) identified geographical areas at risk. The CFIR framework developed within the health care sector provides an opportunity to identify several facilitators and barriers for implementation of IFS interventions within these different types of IFS intervention.

### Types of IFS Intervention

Single individuals are always the target for IFS, but the types of IFS interventions were found to exhibit clear differences in how these individuals were identified and approached. Using one type of intervention in an organisation does not necessarily exclude another as they can be combined. For example, preventive interventions in geographical areas at risk can be done at the same time as there is a program for single individuals who have been identified as being at risk.

#### IFS Intervention: Single Individuals at Risk

In certain municipalities, specific individuals with risk behaviour were identified by social services as appropriate for IFS in their community. These individuals were then approached and evaluated to identify the best solution for their specific needs. For older people with increased risk of fire, a variety of relevant interventions have been implemented in such cases. These include, (1) checklists employed during home visits by healthcare personnel, (2) outreach through specialised information (including printed and internet based), and (3) the installation of technical aids to improve passive and active fire protection. Checklists have been developed typically through collaboration between the fire services and home care professionals. Ideally, the check lists were included in established routines for annual client evaluation to ensure that up to date evaluations were made regularly. Outreach included, e.g. an informative YouTube video tailored to municipalities and citizens alike [46]. Technical aids included stove guards, individualized fire sprinklers and fire safety alarms interconnected with personal safety alarms for older people. In most cases, the cost of such technical aids were covered by the local municipality directly or by means of housing adaptation grants to facilitate their handling and installation. If deemed necessary, a plan for how to increase fire safety could be established and technical systems identified and installed. When using this type of intervention, identifying and approaching a specific individual, the result shows that client confidentiality can become an obstacle that needs to be addressed.

#### IFS Intervention: Identifying Groups at Risk

In certain municipalities, specific groups were identified as at risk, and initial contact was with this group, independent of the needs of the specific individuals within the group. The organizations working at this level typically had a position with a specific responsibility for the IFS intervention, which ensured continuity and facilitated the development of clear best practice and local guidelines. As one participant expressed, should that person change jobs, the implementation of IFS would continue as the position would be filled by a new recruit. In communities with this level of implementation, checklists were developed but were not necessarily connected to home care. General contact could then be supplemented by follow-up phone calls and/or home visits.

At follow-up home visits, the fire service staff would give advice about fire safety and technical solutions, such as stove guards and fire blankets, about routines for replacing batteries in the fire alarm and the risk associated with e.g. storing flammable material, on or in the stove. In cases when older people might have difficulties to reach the fire alarm and testing the battery, the fire services might suggest that children or friends could do this every year, e.g. before Christmas or when visiting in conjunction with a birthday. Due to personal integrity, however, all technical solutions were presented as options together with how to apply for support for installation, but the client was required to be active in applying for implementation of such solutions. No bespoke solution was developed for the individuals rather general recommendations were given for the group.

#### IFS Intervention: Identified Geographical Areas at Risk

At this level of IFS, the rescue services identified geographical areas which were found to exhibit a heightened risk at a general level and try to reach individuals in high risk areas rather than specifically targeting high risk persons. In one example of preventative intervention in a high risk geographical area, representatives from the fire services gave advance notification of home visits in specific areas, asking about home fire safety and offering advice to decrease risk. In one municipality, for example, special attention was placed on apartment buildings in risk areas.

The thinking in this case is that it is effective to target a neighbourhood with low socioeconomic conditions where the problems are “clustered”. On the other hand, this type of intervention tends to miss the most vulnerable individuals. Passive prevention is probably effective in these neighbourhoods, but to reach frail older adults requires cooperation between organisations that are in regular contact with this population specifically with tailored information.

### Facilitators and Barriers for Success for IFS

In this study we identified a number of barriers and facilitators for implementation of IFS interventions in Swedish municipalities. The results show that, as an intervention, IFS has characteristics that potentially facilitate implementation, e.g. a high level of *trialability* and *adaptability*, where the possibility to start small and adapt to local circumstances are frequently used. Another example is that the policy document which is at the heart of IFS is both appreciated and needed, and results show that authority guidelines, and national initiatives play an important role in the implementation of IFS with great symbolic value, in particular for the FRS. However, one of the major barriers to implementation is also linked to the intervention characteristics, i.e. it is unclear where the responsibility for fire protection of vulnerable groups lays. In turn, this effects many aspects of implementation and hinders stakeholders from fully taking responsibility for developing and implementing IFS. This can further be linked to the issue of costs, as the lack of funds is highlighted as a significant barrier to implementation. Unfortunately, the question of cost is closely connected to difficulties for the health care sector and social services to allocation the needed resources in already strained organizations. The question of responsibility for implementation is inextricably tied to the question of cost as without clear resolution of responsibility, the cost is unlikely to be adequately included in annual budgets.

In order to successfully implement IFS, cross-sectoral cooperation between health care and social service organizations and the FRS, is crucial. Although there is general agreement that IFS is needed, collaboration is challenging, both to organize and maintain. According to the FRS, the main obstacle is the lack of resources to prioritize IFS within health care and social services. Preventing the risk of fire, a relatively rare problem, is only one of many important tasks, and is therefore often set aside. Similar problems were identified by Halvorsen et al. [47], who studied fire safety for vulnerable groups in Norway. The results of the present study indicate the need for a deeper mutual understanding of the problem, and to integrate IFS into regular work routines. One suggestion was to include standards for IFS in the procurement phase of home care service planning, to ensure that home care providers planned to include this aspect of home care from the outset. A key facilitator also seems to be designated positions within the FRS, preferably designed to include knowledge and/or experience from health care or social services, to bridge the gap and facilitate cooperation [48].

In the CFIR framework, the inner setting of organisational culture is emphasised as an aspect that effects implementation. Culture is naturally important in all organisations involved in providing IFS; but, as the focus has been on the FRS in the interviews, this will also be the focus of the discussion of organisational culture. In the current case, tradition and norms seem to stand in the way of change. Individualised fire safety is a relatively new task, a task that differs from the traditional role of FRS. Preventative work is not the primary process within the organization, and the FRS has a perceived lack of knowledge in this field. The need for change agents [37] is clearly stated in the results. When someone takes the lead, others can follow. It is not only on an individual level that there is a need for early adopters [42], also on an organisational level this seems beneficial. There is a tradition of experiential learning in the FRS [49], which can be exploited by using lessons learnt from successful implementation in one municipality to another, combining a bottom up approach with experience sharing among and within municipalities. Regarding the process, starting small and gradually implementing IFS is, also a facilitator which potentially builds on this experiential tradition. In contrast, one barrier identified is the lack of follow up to specific activities. A closed feedback loop can be an effective facilitator [39], and developing indicators to measure effects are therefore important. Instead of just focusing on effects regarding fire and injury frequency, indirect measures such as outreach and raising awareness in collaboration organizations can be highlighted as of value.

Lastly, a potential obstacle for implementation, just briefly covered in this study, is the risk prone individual’s perceived lack of need for fire protection, a factor often foreseen in implementation [37]. Vulnerable individuals, most in need for IFS, are often marginalized reflecting underlying social determinants of health [50], also making them prone to other health issues. Therefore, it can be fruitful to apply measures to improve the overall health and safety for vulnerable individuals, e.g. combining fall prevention and fire safety. Collaboration between FRS experts and social services is most likely to result in positive effects on IFS than if one or other organization should endeavor to implement IFS alone [35]. Still, after overcoming e.g. organizational barriers, and identification of risk individuals, the factor of motivation for change still remains [47], an aspects that need to be further explored.

### Methodological Reflections

In this study, matters concerning the implementation of IFS in Swedish municipalities have been qualitatively described. The CFIR framework was a helpful tool in structuring the data and describing the complex challenges and facilitators for implementation. However, the five dimensions and their underlying attributes are interlinked [29] and therefore some of the aspects appear more than once, anticipating to show the matter from different angles. The questions asked were open and not structured by the framework CFIR, which means that data could address additional aspects of the intervention [44], but during the coding process no new category emerged. The CFIR framework was originally developed for implementation of evidence-based health care interventions, but has also been used as a guide to understand other implementation processes (see e.g. [51–53]), reinforcing its broad potential for application.

Due to limitations in time and to the ongoing Covid-19 pandemic, we have focused on the perspective of the FRS. However, in terms of trustworthiness [44], the data was rich and similar issues were addressed in several of the interviews, complemented by secondary data. For example, in greater organizations, the examples given addressed single municipalities in the area, and they were similar to smaller organisations, indicating transferability. Although the major aspects of the problem probably are identified, to get a comprehensive view of the possibilities for implementing IFS among frail older people, the perspective of health care staff and care takers need to be deepened in future studies.

## Conclusions

In this study, similarities and differences between Swedish IFS interventions were described as well as dimensions that offer support for or barriers to implementation of IFS. The general features of the IFS interventions are described as interventions focusing on (1) individuals at risk, (2) groups at risk and (3) geographical areas at risk. Using this division of interventions into three different types, it becomes clear that even municipalities without specific IFS action programs are working on IFS, starting at a general level but nonetheless ending up at an individualised dialogue of fire risk and risk prevention. The results of our study indicates that there is no one size fits all approach to IFS, in particular in light of the complex structure of delegation of authority from national to local level in Sweden, coupled to broad differences in the size and governance of the FRS and home care services at local level.

Despite several barriers, IFS is implemented in many municipalities, and much positive action is being taken to ensure that risk groups, including but not limited to older persons, are given information and support to improve their personal fire safety situation. Should existing barriers be relieved and facilitators be leveraged, even more could be done for these groups in the future. The next step for improving IFS include political support for the interventions, along with a clear definition of responsibility. This latter is closely tied to the question of funding, as until the responsible authority can be determined it is unclear whose budget should include funding provisions. Further, efforts to bridging the gap in knowledge about the target group within FRS is needed, along with improving intersectional cooperation, e.g. appointing positions within the FRS with special knowledge and responsibility for IFS.

Finally, we would like to note that the novel application of the CFIR framework to the field of fire safety has been beneficial in terms of identifying the wide variety of barriers and facilitators to success, but more research is needed to explore how this might be connected to specific strategic choices within an organisation to overcome barriers or leverage facilitators.
